# Seropositive Muscle-Specific Tyrosine Kinase Myasthenia Gravis Presenting as a Late-Onset Isolated Sixth Nerve Palsy: A Case Report and a Brief Review of Subtypes of Myasthenia Gravis

**DOI:** 10.7759/cureus.19701

**Published:** 2021-11-18

**Authors:** Gyusik Park, Hassan Kesserwani

**Affiliations:** 1 Neurology, University of Alabama at Birmingham School of Medicine, Birmingham, USA; 2 Neurology, Flowers Medical Group, Dothan, USA

**Keywords:** neuromuscular junction, autoimmune disease, horizontal diplopia, muscle-specific tyrosine kinase, ocular myasthenia gravis, abducens nerve palsy, musk myasthenia gravis

## Abstract

Autoimmune myasthenia gravis (MG) is a well-characterized post-synaptic disorder of neuromuscular transmission. Immunologically, there is complement activation with autoantibodies binding to the acetylcholine receptor (AChR), leading to cross-linking and internalization of the receptor. The diminished functional clustering leads to impaired folding of the post-synaptic membrane. The antibodies generated by the autoimmune process are directed at the various components of the post-synaptic membrane and its scaffolding, including the AChR, muscle-specific tyrosine kinase (MuSK), low-density lipoprotein receptor-related protein 4 (LRP4), and the other recently described epitopes including the extracellular membrane proteins agrin and collagen Q (ColQ). MuSK MG is phenotypically different from classic AChR-antibody-mediated MG by a more frequent presentation of bulbar weakness, less responsiveness to symptomatic therapy with acetylcholinesterase inhibitors, the absence of a thymoma, and a better therapeutic response to a cluster of differentiation (CD-20) B-cell therapy such as rituximab. The pleiotropic ocular findings of ocular MG include ptosis, fluctuating and variable involvement of cranial nerves III, IV, and VI, pseudo-internuclear ophthalmoplegia (INO), near-complete or complete ophthalmoplegia, and variable gaze palsies. To our knowledge, we present one of the very few reported cases of MuSK MG presenting as isolated sixth nerve palsy. The localization of a sixth nerve palsy with lateral rectus muscle weakness can be due to disease anywhere along its path from the abducens nucleus, coursing at the skull base through Dorello's canal, through the cavernous sinus, and along its path through the superior orbital fissure and into the orbits. A painless sixth nerve palsy should alert the clinician to MuSK-MG as we outline in this case report.

## Introduction

Ocular myasthenia gravis (MG) is characterized by fluctuating weakness of the ocular skeletal muscles which worsens with activity and improves with rest, a cardinal feature of MG. The most frequent presentation is one of ptosis and ocular muscle weakness as outlined above [1]. The pupils are always spared [2]. Spread of weakness to the limbs, bulbar musculature, and the respiratory bellows are known as generalized MG. Bulbar symptoms are more common in elderly males and can include hypophonia, dysarthria, dysphagia, jaw weakness, and neck weakness as in neck flexor or extensor muscle weakness (head drop). Respiratory failure due to MG is known as a myasthenic crisis and can be heralded by bulbar weakness, necessitating hospital admission, ventilatory support, and therapy with plasma exchange (PEX) or intravenous immunoglobulin (IVIg) [3].

Around 80% of cases of MG are caused by autoantibodies directed against AChR. MuSK antibodies account for 4% of cases, LRP4 antibodies account for 2% of cases, and 5% are seronegative [4]. Among AChR-negative MG, 30%-40% of cases carry MuSK antibodies [5]. MuSK- and AChR-negatives cases are labeled as a double negative. LRP-4-MG is found in a small proportion of double negative cases [6]. Triple-negative cases are seronegative for MuSK, AChR, and LRP-4 antibodies. Only a few such patients harbor agrin and ColQ antibodies [7]. These proteins interact on the post-synaptic membrane by forming a tetrameric complex between MuSK and LRP-4. Phosphorylation of the MuSK-LRP-4 complex enables AChR clustering which enhances neuromuscular transmission.

## Case presentation

We present the case of a 74-year-old man who presented with a sudden onset of painless horizontal diplopia worse with a left-directed gaze. The double vision was aborted by occluding either eye. With eye-straining, he developed a dull bitemporal headache. He denied any vision loss, dysarthria, dysphagia, chewing difficulty, neck weakness, or breathing difficulty. A visit to the ophthalmologist confirmed a normal ocular funduscopic examination and left sixth nerve palsy. His condition remained static until he presented to the neurology clinic one month later.

Past medical history was significant for bilateral below-knee amputations due to peripheral vascular disease secondary to smoking, which he quit many years ago. Otherwise, he was in relatively good health. He denied the use of any medications including antiplatelet therapy, cilostazol, statins, or anti-hypertensives. He also denied any constitutional symptoms such as fatigue, myalgias, muscle wasting, fevers, or joint pains.

**Figure 1:**
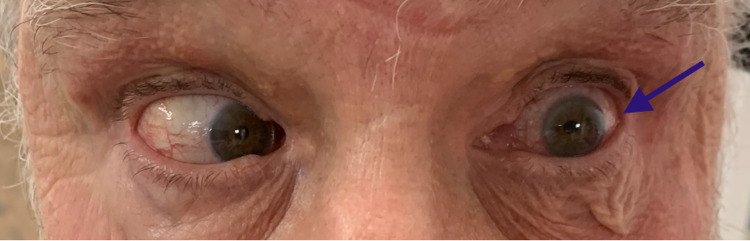
Left sixth nerve palsy showing the weakness of left lateral rectus muscle (blue arrow)

On examination, the patient appeared alert, oriented, well-nourished, and in no apparent distress. Blood pressure was recorded at 134/80 mmHg, a pulse at 64 beats per minute, and respiratory rate at 12 per minute. The patient refused to be weighed. His speech was of normal tone, volume, and prosody without any hint of dysarthria or fatigability. Cranial nerve examination revealed no facial weakness with symmetric smile, intact whistling, and no difficulty blowing his cheeks. There was an obvious left lateral rectus paresis upon assuming left gaze (Figure 1).

Vertical gaze was unaffected, and the pupils were spared. Masseter, genioglossus, and pterygoid function were preserved with intact jaw closure, deviation, and opening. The gag reflex was brisk. Neck flexion and extension showed adequate movement against resistance. Power in the arms was graded at 5/5 in all muscle groups with the medical research council (MRC) grading scale. Bilateral below-knee-amputation was noted, with preservation of bilateral hip flexion, adduction, and abduction. Deep tendon reflexes in the upper extremities were preserved with normal finger-to-nose coordination.

A magnetic resonance imaging (MRI) of the brain with and without gadolinium enhancement revealed no lesion of the brainstem or cavernous sinus, and magnetic resonance angiography (MRA) revealed no cavernous sinus aneurysm. An MG panel for AChR modulating, binding, and blocking antibodies was negative. Striational antibodies were negative. A MuSK-antibody titer was high at 1.6 units per milliliter (mL); positive is 1.0 or higher. A sedimentation rate was normal. Based upon the negative MRI and MRA of the brain findings, the ocular manifestations, positive MuSK serology, a diagnosis of MuSK-ocular MG was made. A repetitive nerve stimulation (RNS) test and single-fiber electromyography (SFEMG) test were not scheduled. A trial of pyridostigmine at a dose of 60 milligrams (mg) three times daily was ineffective. The patient opted against therapy with prednisone and/or rituximab, and he preferred wearing an occlusive eye patch. The patient was advised about the potential for bulbar weakness and myasthenic crisis.

## Discussion

Epidemiologically, MuSK-MG is usually a disease of young females. However, as our case highlights, MuSK-MG is not uncommon in the elderly especially when the semiology does not conform to the usual characteristics of AChR-MG. MuSK-MG has several unique defining features that include a relatively acute bulbar-onset with a lack of fluctuations that may herald a myasthenic crisis, with it being more frequent than in classic AChR-MG. The clinical features of AChR, MuSK, and LRP-4 related MG are summarized below in Table 1 [8]. Other unique features of MuSK-MG include neck extensor weakness (head drop) being far more common than neck flexor weakness and muscle atrophy involving the tongue, facial muscles, shoulder girdles, and paraspinal muscles. Electrophysiologically, these atrophied muscles demonstrate myopathic units with fatty infiltration on muscle biopsy. We also emphasize the absence of thymic pathology as noted in Table 1 [9].

**Table 1:** Clinical characteristics of three subtypes of myasthenia gravis AChR = acetylcholine receptor, MuSK = muscle-specific tyrosine kinase, LRP-4 = lipoprotein receptor-related protein, IVIg = intravenous immunoglobulin, PEX = plasma exchange

	Age at onset	Sexual dimorphism	Clinical semiology	Thymic pathology	Acetylcholinesterase inhibitor responsiveness	Immunotherapy
AChR	Early-onset/Later onset (> 50 years)	Early-onset (female)/Later onset (male)	Variable (ocular, generalized, and crisis)	Thymic hyperplasia, thymoma	Effective	Prednisone, azathioprine, IVIg, PEX
MuSK	Third decade	Usually female	Bulbar involvement, muscle atrophy, head drop more common	No thymic pathology	No response	IVIg, PEX, rituximab
LRP-4	Variable	Usually female	Variable	Rarely hyperplastic thymus	Effective	Prednisone, azathioprine, IVIg, PEX

The diagnosis of MuSK-MG can be challenging as this subset of patients are usually resistant to acetylcholinesterase inhibitors. The RNS test has lower sensitivity, and SFEMG is usually not available except in neuromuscular clinics. Nevertheless, an electrodecremental response, when present, is usually downward sloping and not parabolic as in AChR-MG. Despite low sensitivity, serological testing is of paramount importance as the specificity is very high. Cell-based assays (antibodies directed against surface antigens on cells) have improved sensitivity when compared to radioimmunoassay, especially in double-negative cases [10]. However, it has been our clinical experience that there is enough data in the atypical clinical profile, serology, and electrophysiology to allow the clinician to zero in on the diagnosis with relative ease.

Therapeutically, MuSK-MG has a relatively well-established treatment algorithm. Therapy should be initiated with steroids and IVIg cover. Respiratory compromise should necessitate hospital admission for IVIg or PEX and respiratory support. Maintenance therapy consists of dual immunosuppressive therapy with the lowest possible long-term effective dose of prednisone and CD-20 B-cell-based intravenous therapy with rituximab [11]. In our experience, this paradigm is therapeutically effective.

A case of MG patient presenting with isolated abducens nerve palsy has been reported once before [12]. Our case represents the second such case. With this finding, we propose that isolated abducens nerve palsy may be associated with MuSK-MG as part of the differential diagnosis, and future case reports and series may confirm this association.

## Conclusions

MuSK-MG is a more severe and rarer subtype of MG that presents with unique clinical features that distinguish it from classic AChR-MG. In this case report, we expand the phenotypic range and presentation of MuSK-MG and suggest that it include isolated abducens nerve palsy. The high prevalence and therapeutic implications of MG warrant future endeavors in further characterizing MG subtypes.
